# Modification of the Interface Nanostructure and Magnetic Properties in Nd-Fe-B Thin Films

**DOI:** 10.1186/s11671-016-1227-x

**Published:** 2016-01-19

**Authors:** Kunihiro Koike, Takanao Kusano, Daisuke Ogawa, Keisuke Kobayashi, Hiroaki Kato, Mikihiko Oogane, Takamichi Miyazaki, Yasuo Ando, Masaru Itakura

**Affiliations:** Department of Applied Mathematics and Physics, Yamagata University, Yonezawa, 992-8510 Japan; Department of Applied Physics, Tohoku University, Sendai, 980-8579 Japan; Department of Applied Science for Electronics and Materials, Kyushu University, Kasuga, 816-8580 Japan

**Keywords:** Nd_2_Fe_14_B thin film, Nd overlayer effect, Interface, Coercivity

## Abstract

The effects of Nd_2_Fe_14_B grain size and Nd coating on the coercivity in sputter-deposited Nd-Fe-B/Nd thin films have been investigated in order to gain an insight into the coercivity mechanism of Nd-Fe-B magnets. Highly textured Nd_2_Fe_14_B particles were grown successfully on the MgO(100) single-crystal substrate with the Mo underlayer. As the Nd-Fe-B layer thickness *t*_NFB_ was decreased from 70 to 5 nm, the coercivity *H*_*c*_ increased gradually from 6.5 to 16 kOe. By depositing the Nd overlayer onto these films and post-annealing at 500 °C, the *H*_*c*_ value further increased from 17.5 kOe (*t*_NFB_=70 nm) to 26.2 kOe (*t*_NFB_=5 nm). The amount of *H*_*c*_ increase by the combination of the Nd coating and post-annealing was about 10 kOe irrespective of the *t*_NFB_ value. These results therefore suggest an independence of size and interface effects on the coercivity of Nd-Fe-B magnets.

## Background

Achieving higher coercivity in sintered Nd-Fe-B magnets without using rare elements such as Dy and Tb is one of the most crucial and urgent subjects [1–3], in view of the rapidly growing demand for high-power motors such as those in electric vehicles. At least two approaches have been reported so far to increase coercivity *H*_*c*_ in sintered Nd-Fe-B magnets. First one is the grain size refinement. The *H*_*c*_ value is known to increase gradually with decreasing average grain size *d*_grain_ [4, 5]. Une and Sagawa recently reported [6] the record value of *H*_*c*_ ∼20 kOe for the sample with *d*_grain_∼ 1 *μ*m. The second approach is the interface control [7–9], in which the appropriate heat treatment at 500 ∼ 600 °C gives rise to a smoother core-shell structure between the Nd_2_Fe_14_B core and Nd-rich shell, resulting in an optimum coercivity value. Recent study of the polished surface of sintered Nd-Fe-B magnets [10, 11] claims that an existence of the fcc-type Nd oxide is crucial for the *H*_*c*_ recovery. In order to investigate the relation between such interface state and the coercivity in sintered Nd-Fe-B magnets, we chose to construct a model system by a thin-film technique. We reported that the *H*_*c*_ increases from 11 to 14 kOe when the highly oriented Nd_2_Fe_14_B grains with the lateral dimension of about 2 ∼ 3 *μ*m were coated by the Nd overlayer and annealed at 500 °C [12]. Transmission electron microscopy (TEM) and X-ray investigations indicate the existence of the Nd oxide also in this sample. The important geometric difference between our model system and the real sintered Nd-Fe-B magnets is that the Nd_2_Fe_14_B core is not fully wrapped by the Nd-rich shell, but only the top and side surfaces are in contact with the Nd-rich phase in the former. This suggests the importance of side surfaces of Nd_2_Fe_14_B structure which is parallel to the *c*-axis [13]. However, it is difficult to evaluate such an interface contribution to the coercivity in the previous report [12], since the final *H*_*c*_ value and the amount of *H*_*c*_ increase are not so large enough. There is another question as to whether the size and interface effects on the coercivity are independent or not. In this work, we fabricated higher quality Nd-Fe-B/Nd films by using single-crystal substrates with different grain sizes and investigated the size and interface dependence of the coercivity systematically.

## Methods

Nd_2_Fe_14_B/Nd films were deposited by using the UHV helicon sputtering system onto MgO(100) single-crystal substrate. The base pressure of the sputtering chamber was less than 5 ×10^−8^ Pa. The films have the forms of MgO(100)/Mo(20 nm)/Nd-Fe-B(5 nm ≤*t*_NFB_≤ 70 nm)/Nd(*t*_Nd_= 0 or 60 nm)/Mo(10 nm). As the sputtering targets, we used the 3N-purity ternary alloy of Nd_17_Fe_70_B_13_, Nd metal with 2.5N purity, and Mo metal with 4N purity. The cosputtering of the Nd_17_Fe_70_B_13_ and Nd targets was done in order to adjust a composition of the Nd-Fe-B layer. The Ar gas pressure during the deposition of the Mo, Nd, and Nd-Fe-B layers was 0.07, 0.07, and 0.2 Pa, respectively. We heated the substrate during the deposition of the Mo underlayer at $T_{\mathrm {s}^{Mo}} =$ 300 °C, and then post-deposition annealing was done at $T_{\mathrm {a}^{Mo}} =$ 700 °C. The Nd-Fe-B main layer and overlayers of Nd and Mo were deposited successively at substrate temperatures of $T_{\mathrm {s}^{NFB}} =$ 600 °C and $T_{\mathrm {s}^{Nd}} = T_{\mathrm {s}^{Mo}} =$ 50 °C, respectively. In some films, the post-annealing was done in situ at *T*_a_= 500 °C for 40 min before depositing the Mo overlayer.

Above conditions for the Mo underlayer are the ones optimized according to the several reports on the high coercivity of Nd-Fe-B films [14–16]. As for the post-annealing procedure for the Mo underlayer deposited on the heated substrate, we already published it in separate papers [17, 18].

Magnetization curves at room temperature were measured by using SQUID magnetometer in fields of up to 50 kOe. Crystal structure and orientation of the films were evaluated by using X-ray diffraction (XRD) and pole-figure apparatus with the Cu- *Kα* radiation. X-ray reflectivity (XRR) measurements were done to evaluate the film thickness. Atomic force microscopy (AFM) and scanning electron microscopy (SEM) systems were used to observe the surface morphology of the films, in which the average diameter of Nd_2_Fe_14_B grains in lateral direction was estimated by using particle analysis software, ImageJ. The microstructure and composition of films were characterized by using TEM, nano-beam electron diffraction (NBD), and energy-dispersive spectroscopy (EDS) techniques.

## Results and Discussion

**Fig. 1:**
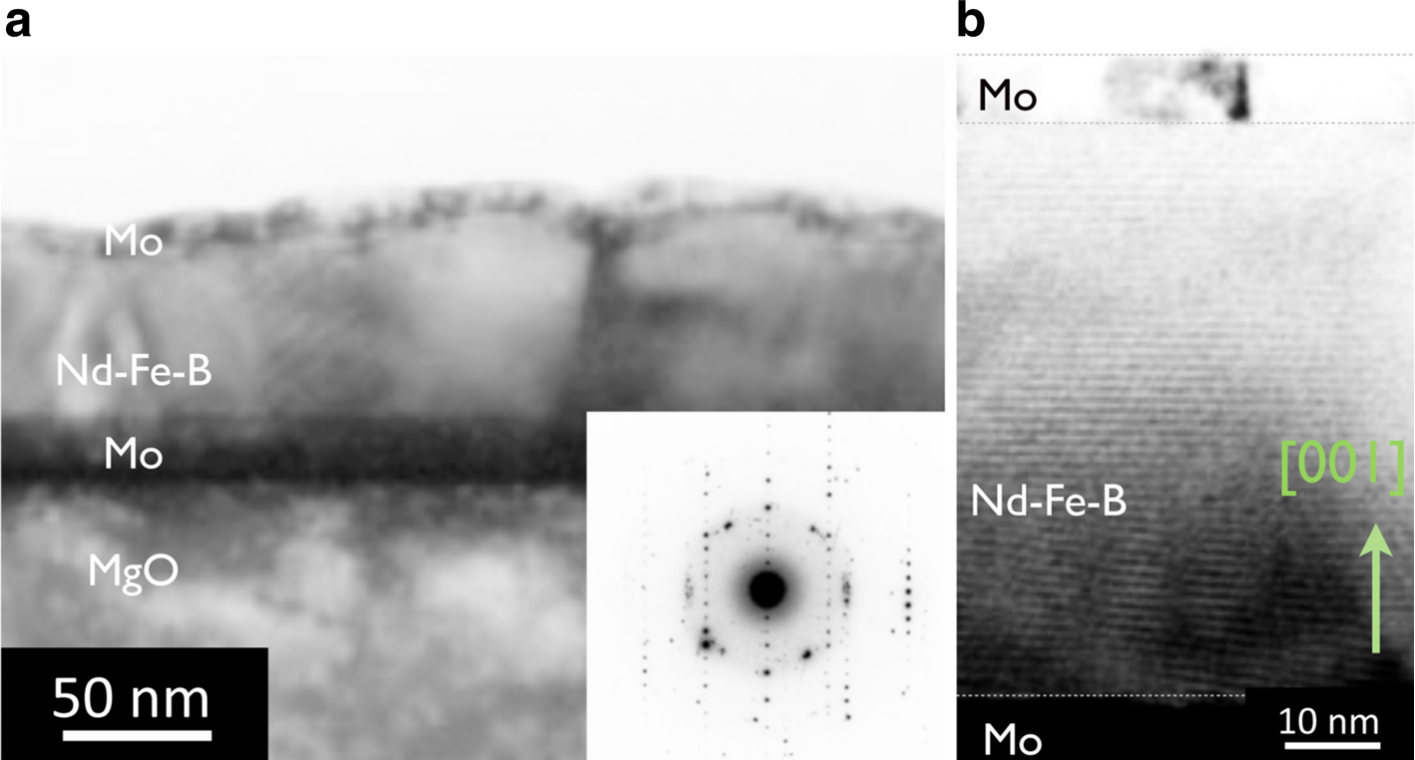
Cross-sectional TEM images and the NBD pattern for the Nd-Fe-B(*t*_NFB_ = 70 nm) film. **a** Cross-sectional TEM images for the as-deposited Nd-Fe-B film. **b** Cross-sectional TEM images with different magnifications for the Nd-Fe-B film. The inset in **a** shows the NBD pattern for a position in the Nd-Fe-B layer

Cross-sectional TEM images with the NBD pattern for the film with *t*_NFB_= 70 nm and *t*_Nd_= 0 are shown in Fig. 1. Both the lattice fringe of Nd_2_Fe_14_B structure in the magnified scale image and the multiple scattering spots in the NBD pattern indicate the preferential texturing of the *c*-axis of the Nd_2_Fe_14_B crystallites normal to the film plane.

**Fig. 2:**
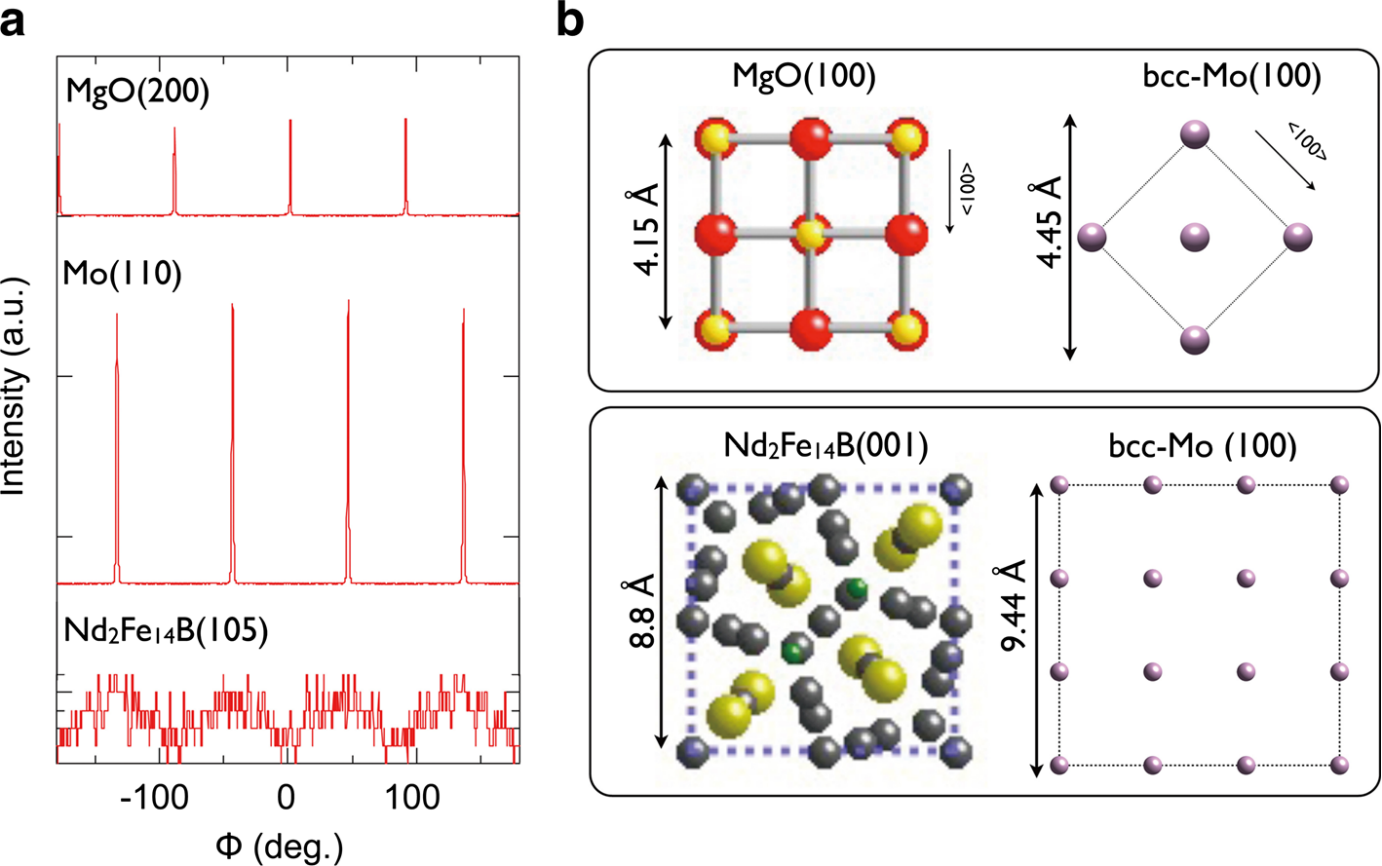
*ϕ*-scan representations of X-ray pole-figure measurements for the as-deposited Nd-Fe-B(*t*_NFB_=70 nm) film. **a** *ϕ*-scan representations of X-ray pole-figure measurements for MgO, Mo, and Nd_2_Fe_14_B layers in as-deposited MgO/Mo/Nd-Fe-B(*t*_NFB_=70 nm)/Mo film. **b** Estimated epitaxial relationships between the layers

Figure 2 shows the *ϕ*-scan representations of X-ray pole-figure measurements for the (200) plane of the MgO single-crystal substrate, (110) plane of the Mo underlayer, and (105) plane of the Nd_2_Fe_14_B layer. Clear fourfold symmetry peaks were observed for MgO and Mo layer in which the peak positions are shifted each other by 45°. Although weak, we did find the fourfold symmetry peaks also for the Nd_2_Fe_14_B layer in the same positions as those for the Mo underlayer. These results thus suggest that the Nd_2_Fe_14_B(001) crystallites are epitaxially grown with the relationships of MgO(100) [110]∥Mo(100)[100], and Mo(100) [100]∥Nd_2_Fe_14_B(001)[100], as shown schematically in Fig. 2b. These relationships are in accordance with those for MgO(100)/Cr/Ta(100)/Nd_2_Fe_14_B [19].

**Fig. 3:**
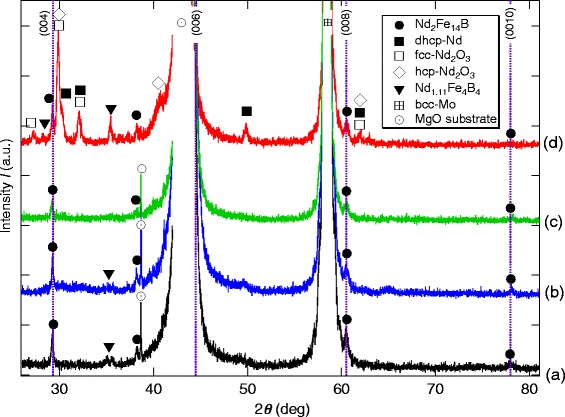
XRD patterns for the Nd-Fe-B(*t*_NFB_=70 nm) films. XRD patterns for the films with *t*_NFB_=70 nm **a** *t*_Nd_= 0, as-deposited; **b** *t*_Nd_=0, post-annealed; **c** *t*_Nd_=60 nm, as-deposited; **d** *t*_Nd_=60 nm, post-annealed

**Fig. 4:**
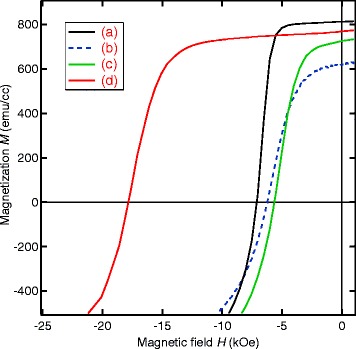
Demagnetization curves for the Nd-Fe-B(*t*_NFB_=70 nm) films. Demagnetization curves for the four films **a** ∼**d** given in Fig. 4 with *t*_NFB_=70 nm. External magnetic fields were applied normal to the film plane. No correction for the demagnetizing field has been made

Figure 3 shows the XRD patterns for the four films with *t*_NFB_=70 nm fabricated by changing a combination of the two parameters; the presence/absence of the Nd overlayer and with/without the post-deposition annealing at *T*_a_= 500 °C. In addition to the strong peaks from MgO substrate and Mo layers, we do observe in all the films the diffraction peaks from the (004), (006), (008), and (0010) planes of the tetragonal Nd_2_Fe_14_B phase, which indicate that the *c*-axis of the Nd_2_Fe_14_B crystallites aligns perpendicular to the film plane. This result is inconsistent with the TEM and pole-figure data shown in Figs. 1 and 2. In the case of the post-annealed film with the Nd overlayer, as shown in Fig. 4d, we observe extra peaks from not only the dhcp-Nd metal phase but also the Nd oxide phase such as metastable fcc-Nd_2_O_3_(space group: $T_{\mathrm {h}^{7}}$-$Ia\overline {3}$) and the stable hcp-Nd_2_O_3_($D_{\mathrm {3d}^{3}}$-$P\overline {3}m1$). Considering that no such extra peaks were observed in the as-deposited film with the Nd overlayer shown in Fig. 3c, these Nd oxide phases were formed during the post-annealing in which the oxygen would originate mainly in the Nd targets.

Figure 4 shows the demagnetization curves for the four 70-nm-thick films given in Fig. 3. While the *H*_*c*_ values for the three films (a) ∼ (c) fall within the range from 6 to 7 kOe, much larger *H*_*c*_ of 17.5 kOe was recorded for the film (d) in which we have deposited the Nd overlayer and made the post-annealing. This result clearly indicates that both the Nd overlayer and the post-annealing are indispensable for the high coercivity in the present system.

It should be noted that the magnetization values for the three films (b), (c), and (d) are slightly smaller than that for the as-deposited Nd-Fe-B film (a). However, owing to the difficulty in precise estimation of the sample volume, it is not easy to discuss whether these changes in magnetization are significant or not.

**Fig. 5:**
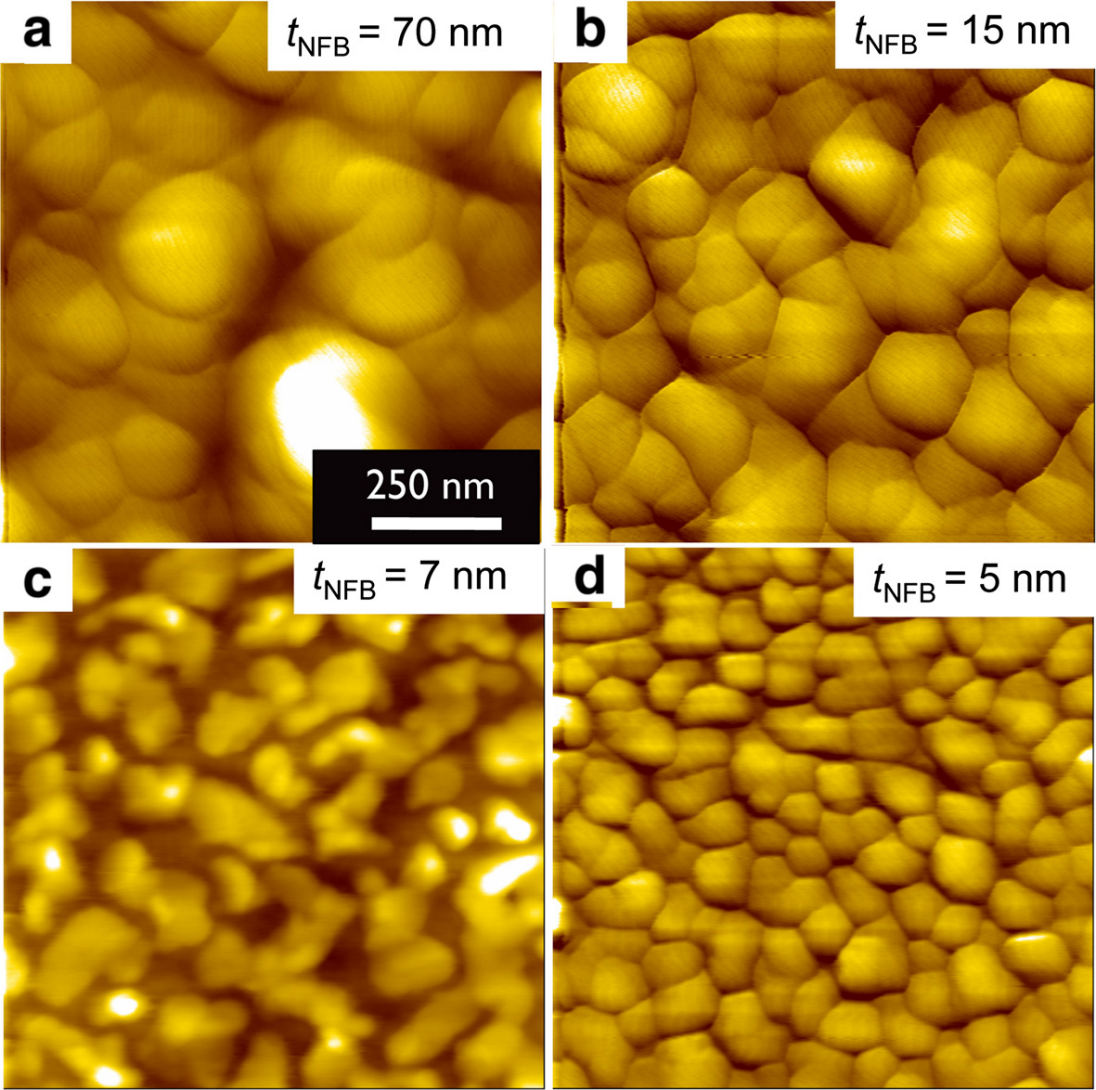
AFM images for the Nd-Fe-B films with various Nd-Fe-B layer thickness *t*_NFB_. AFM images for the as-deposited Nd-Fe-B films with *t*_Nd_=0 and *t*_NFB_=**a** 70 nm, **b** 15 nm, **c** 7 nm, **d** 5 nm

Figure 5 shows the surface images observed by AFM for the MgO(100)/Mo/Nd-Fe-B/Mo films with various Nd-Fe-B layer thickness *t*_NFB_. Surface morphology suggesting an assembly of particles is seen in which the typical lateral dimension varies from about 300 nm to less than 10 nm, with decreasing *t*_NFB_ values.

Although we should keep in mind that these values of lateral dimension do not always correspond to grain size, these data suggest that the lateral grain size decreases with decreasing nominal thickness. We have also performed the SEM measurements to evaluate the microstructure in these films and found the results which support the speculation above.

**Fig. 6:**
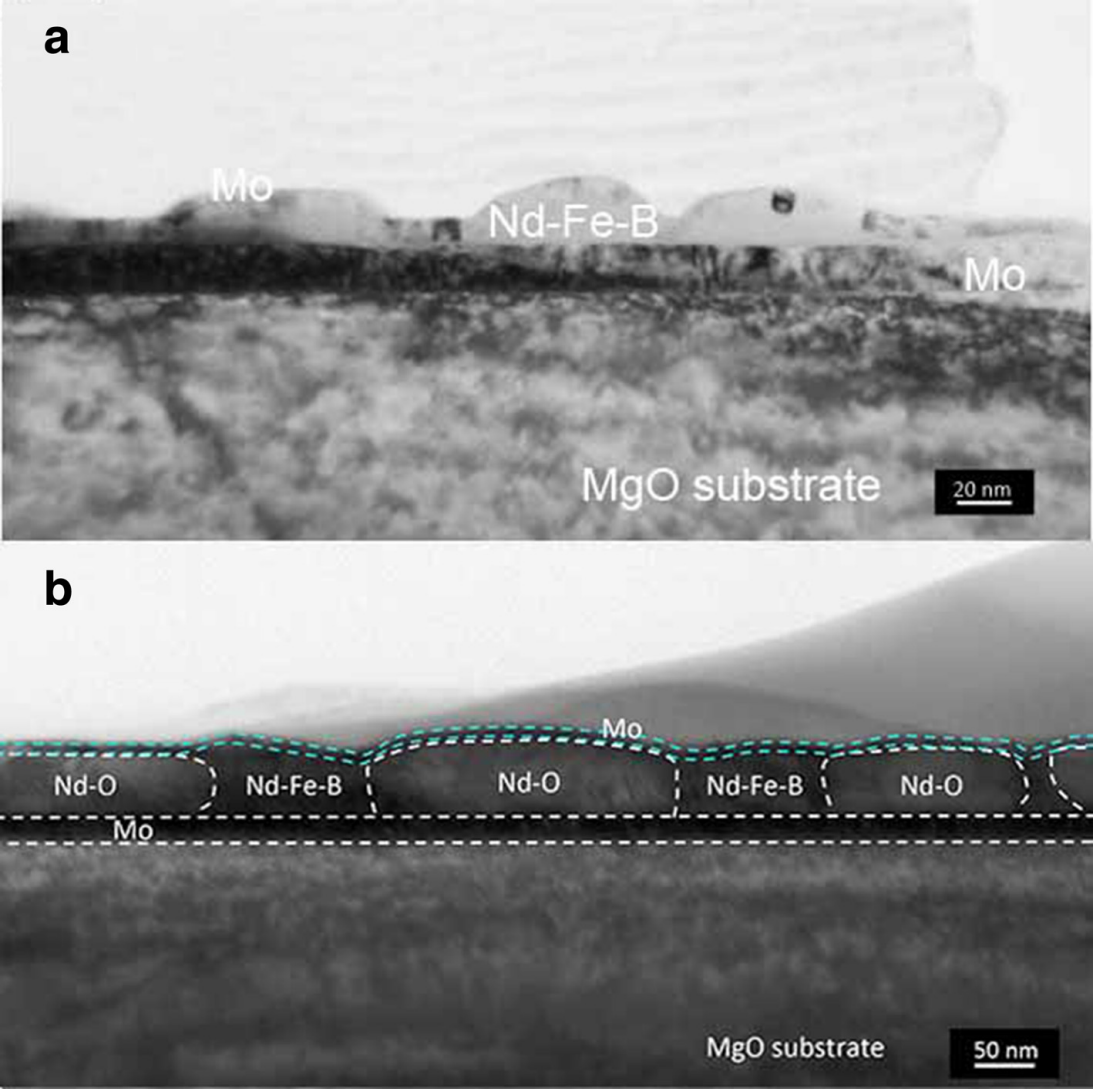
Cross-sectional TEM images for the Nd-Fe-B films with *t*_NFB_=5 nm. Cross-sectional TEM images for the **a** as-deposited Nd-Fe-B (*t*_NFB_=5 nm) and **b** annealed Nd-Fe-B (*t*_NFB_=5 nm)/Nd(*t*_Nd_=60 nm) films. Broken lines in **b** are drawn according to the EDS assignments of the chemical composition

When the value of *t*_NFB_ is less than 10 nm, we can notice the dark color region surrounding each particle, which suggests that the Nd_2_Fe_14_B particles are separated with each other. Such island-like surface morphology is similar to that reported for the MgO(100)/Cr/Mo/Nd-Fe-B/Mo films [20]. Cross-sectional TEM images for two films with *t*_NFB_=5 nm, one for the as-deposited Nd-Fe-B and another for the post-annealed Nd-Fe-B/Nd, are shown in Fig. 6. These cross-sectional images exhibited the island-like structure of the Nd_2_Fe_14_B grains with the lateral dimension of about 60 nm.

In the case of post-annealed Nd-Fe-B/Nd film, the Nd and/or Nd-O locate mainly in a valley among isolated Nd_2_Fe_14_B grains. This morphology is in marked contrast to that reported in [21], in which Nd-O particles locate within the Nd_2_Fe_14_B grains.

**Fig. 7:**
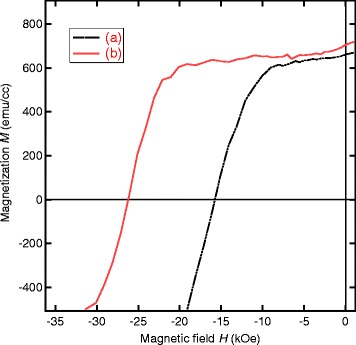
Demagnetization curves for the Nd-Fe-B films with *t*_NFB_=5 nm. Demagnetization curves for the **a** as-deposited Nd-Fe-B (*t*_NFB_=5 nm) and **b** annealed Nd-Fe-B (*t*_NFB_=5 nm)/Nd(*t*_Nd_=60 nm) films given in Fig. 6. External magnetic fields were applied normal to the film plane. No correction for the demagnetizing field has been made

Demagnetization curves for the 5-nm-thick films shown in Fig. 6 are given in Fig. 7. We observed the highest *H*_*c*_ value of 26.2 kOe in the post-annealed Nd-Fe-B/Nd film, which is about 10 kOe larger than that of the as-deposited Nd-Fe-B films. Although not given in this figure, similar dependence of *H*_*c*_ on the two parameters was found in 5-nm-thick films. Namely, the *H*_*c*_ values for the films without the Nd overlayer and the film with Nd overlayer but without annealing were in the range from 15 to 17 kOe.

**Fig. 8:**
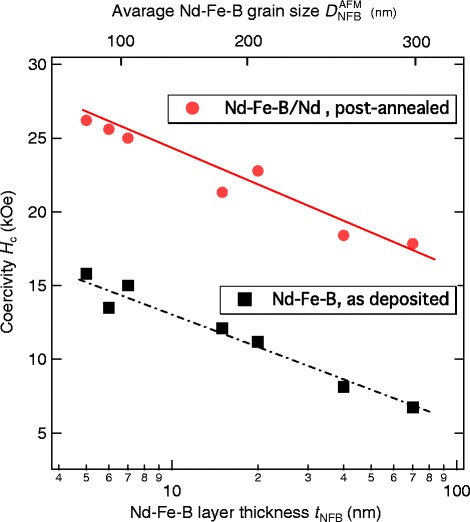
Coercivity *H*_*c*_ of the Nd-Fe-B films as a function of the Nd-Fe-B layer thickness *t*_NFB_. *H*_*c*_ as a function of Nd-Fe-B layer thickness *t*_NFB_ and the average grain size along the lateral direction $D_{\text {NFB}^{AFM}}$, for the Nd-Fe-B/Nd films after post-annealing (*filled circle*) and the as-deposited Nd-Fe-B films (*filled square*)

We then plotted in Fig. 8 the *H*_*c*_ value as a function of Nd-Fe-B layer thickness *t*_NFB_, for the two series of films. Also labelled in the top axis is the average lateral grain size $D_{\text {NFB}^{AFM}}$, which we estimated from the AFM images given in Fig. 5, assuming that each particle-like roughness represents the single Nd_2_Fe_14_B grain. In the case of the films without the Nd overlayer and without the post-annealing, the coercivity exhibited a gradual increase behavior from 6.5 to 16 kOe, as the *t*_NFB_ was decreased from 70 to 4 nm.

It is well known that the coercivity increases with decreasing grain size in sintered Nd-Fe-B magnets [22, 23]. Although the shape of Nd_2_Fe_14_B grains are not spherical but oblate in the present films, it is no wonder that we observed a similar size dependence of coercivity in our films. Recent experimental report for the hot-deformed magnets [24] also exhibited the similar behavior to the present results.

In a series of films with the Nd overlayer after the post-annealing, the *H*_*c*_ value also increased monotonically with decreasing *t*_NFB_ and exhibited the maximum of *H*_*c*_=26.2 kOe for *t*_NFB_=5 nm. It should be noted that the amount of *H*_*c*_ increase by the Nd coating is about 10 kOe irrespective of *t*_NFB_ value.

This fact therefore suggests that the grain size and the Nd coating have an independent influence on the coercivity, since the AFM images shown in Fig. 3 suggest that the average Nd-Fe-B grain size $D_{\text {NFB}^{AFM}}$ decreases with decreasing *t*_NFB_. Of course, we need to take account of the shape of the Nd-Fe-B grain in the present films which would be far from isotropic, in order to discuss the size dependence of *H*_*c*_ more precisely.

It is interesting to note that the *H*_*c*_ value continues to increase with decreasing size, although even the largest dimensions in the films in Fig. 8 are already smaller than the critical size for the single domain in the Nd_2_Fe_14_B phase.

**Fig. 9:**
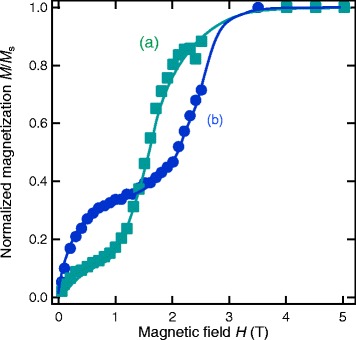
Initial magnetization curves for the Nd-Fe-B (*t*_NFB_=5 nm) and Nd-Fe-B (*t*_NFB_=5 nm)/Nd *t*_Nd_=60 nm) films. Initial magnetization curves for the **a** as-deposited Nd-Fe-B (*t*_NFB_=5 nm) and **b** annealed Nd-Fe-B (*t*_NFB_=5 nm)/Nd *t*_Nd_=60 nm) films given in Fig. 7

In order to obtain further information of the coercivity mechanism, we performed the initial magnetization curve measurements. The results for the two *t*_NFB_=5 nm films given in Fig. 7 are shown in Fig. 9. We observed the double-step behavior of initial curve for both films. But, the post-annealed Nd-Fe-B/Nd film has a much higher field for the second step occurrence. It is interesting to note that the field of inflection point for the second step is nearly equal to the coercivity value in both films. Similar behavior was reported for Nd-Fe-B/FeCo multilayer system [25].

Results given in Fig. 8 suggest that a proper interface state is extremely important to achieve high *H*_*c*_ not only in larger grain system but also in smaller particle one. Preliminary TEM observations for films with the Nd overlayer after the post-annealing as shown in Fig. 6b showed isolated Nd_2_Fe_14_B grains between the Nd oxide grains. This fact would be related to the microstructure of the Nd-Fe-B/Nd system in previous reports [11, 26], in which the coercivity change was discussed in relation to the presence of the fcc-type Nd oxide phase within the Nd overlayer in the close proximity of the Nd_2_Fe_14_B grains.

## Conclusions

We fabricated highly textured Nd_2_Fe_14_B films with and without the Nd overlayer and investigated their magnetic properties systematically. As shown in Fig. 8, the parallel behavior of the two *H*_*c*_ vs *t*_NFB_ lines for as-deposited Nd-Fe-B and post-annealed Nd-Fe-B/Nd films enabled us to suggest that the grain size and the interface state are independent parameters for the coercivity. This work will thus give an insight into the approaches [27] to the fabrication of higher coercivity Nd-Fe-B magnets by reducing the grain size. High-resolution TEM studies are now in progress in order to investigate the interface state and to check whether there is a structural difference between the side and top interfaces of the Nd_2_Fe_14_B grains or not.

## References

[1] Sugimoto S (2011). Current status and recent topics of rare-earth permanent magnets. J Phys D Appl Phys.

[2] Gutfleisch O, Willard M, Chen EBC, Sankar S, Liu J (2011). Magnetic materials and devices for the 21st century:stronger, lighter, and more energy efficient. Adv Mater.

[3] Hirosawa S (2015). Current status of research and development toward permanent magnets free from critical elements. J Magn Soc Jpn.

[4] Muller KH, Eckert D, Handstein A, Nothnagel P, Schneider J (1991). Material structure and coercivity of sintered Nd-Fe-B type magnets. J Magn Magn Mater.

[5] Pan L, Cao D, Jing P, Wang J, Liu Q (2015). A novel method to fabricate CoFe2O4/SrFe12O19 composite ferrite nanofibers with enhanced exchange coupling effect. Nanoscale Res Lett.

[6] Une Y, Sagawa M (2012). Enhancement of coercivity of Nd-Fe-B sintered magnets by grain size reduction. J Japan Inst Metals.

[7] Zhou G, Fu S, Sun X, Chuang Y (1990). Influence of annealing on the magnetic properties and microstructure of nd-fe-b based magnets. J Appl Phys.

[8] Vial F, Joly F, Nevalainen E, Sagawa M, Hiraga K, Park K (2002). Improvement of coercivity of sintered NdFeB permanent magnets by heat treatment. J Magn Magn Mater.

[9] Liu M, Jin T, Hao L, Cao J, Wang Y, Wu D, Bai J, Wei F (2015). Effects of Ru and Ag cap layers on microstructure and magnetic properties of FePt ultrathin films. Nanoscale Res Lett.

[10] Hirosawa S, Tokuhara K, Sagawa M (1987). Coercivity of Surface Grains of Nd-Fe-B Sintered Magnet. J J Appl Phys.

[11] Fukagawa T, Hirosawa S (2008). Influence of Nd/Nd2Fe14B interface microstructure on the coercivity of surface Nd2Fe14B grains in Nd-sputtered Nd–Fe–B sintered magnets. Scripta Mater.

[12] Koike K, Igarashi S, Yamaguchi K, Kusano T, Miyazaki T, Ogawa D, Akiya T, Adachi Y, Kato H (2010). Effect of rare-earth overlayer on the coercivity in sputter-deposited Nd-Fe-B films Effect of rare-earth overlayer on the coercivity in sputter-deposited Nd-Fe-B films. J Phys Conf Series.

[13] Makita K, Yamashita O, Kato H (2002). Boundary Structure and the Local Crystalline Electric Field of Nd-Fe-B Sintered Magnets. J Mag Soc Jpn.

[14] Keavney D, Fullerton E, Pearson J, Bader S (1997). High-coercivity, c-axis oriented Nd2Fe14B films grown by molecular beam epitaxy. J Appl Phys.

[15] Chen S, Liu W, Chen C, Zhang Z (2005). Effects of buffer layer and substrate temperature on the surface morphology, the domain structure and magnetic properties of c-axis-oriented Nd2Fe14B films. J Appl Phys.

[16] Cui W, Zhang S, Liu W, Ma X, Yao Q, Zhang Z, Zhang X (2008). Anisotropic behavior of exchange coupling in textured Nd2Fe14B/a-Fe multilayer films. J Appl Phys.

[17] Koike K, Umezawa J, Ishikawa H, Ogawa D, Mizuno Y, Kato H, Miyazaki T, Ando Y (2014). Effect of Dy/Nd double layer on coercivity in Nd-Fe-B thin films. J Appl Phys.

[18] Koike K, Ishikawa H, Ogawa D, Kato H, Miyazaki T, Ando Y, Itakura M (2015). Coercivity enhancement in La coated Nd-Fe-B thin films. Phys Procedia.

[19] Kwon A, Hannemann U, Neu V, Fähler S, Schultz L (2005). Microstructure and magnetic properties of highly textured Nd–Fe–B films grown on Ta(100). J Magn Magn Mater.

[20] Sato T, Oka N, Ohsuna T, Kaneko Y, Suzuki S, Shima T (2011). Enhancement of coercivity for Nd-Fe-B thin films by the infiltration of Nd-Cu alloy cap layer. J Appl Phys.

[21] Chen S, Zheng J-G, Liu W, Zhang Z (2007). Structure and magnetic properties of high-energy product Nd-Fe-B/Nd-O thin films. J Phys D: Appl Phys.

[22] Ramesh R, Srikrishna K (1988). Magnetization Reversal in Nucleation Controlled Magnets Part 1 : Theory. J Appl Phys.

[23] Hono K, Sepehri-Amin H (2012). Strategy for high-coercivity Nd–Fe–B magnets. Scripta Mater.

[24] Liu J, Sepehri-Amin H, Ohkubo T, Hioki K, Hattori A, Schrefl T, Hono K (2015). Grain size dependence of coercivity of hot-deformed Nd–Fe–B anisotropic magnets. Acta Mater.

[25] Cui W, Takahashi Y-K, Hono K (2012). Nd2Fe14B/FeCo Anisotropic Nanocomposite Films with a Large Maximum Energy Product. Adv Mat.

[26] Matsuura M, Sugimoto S, Goto R, Tezuka N (2009). Interfacial state and magnetic properties of Nd-Fe-B/Nd thin films. J Appl Phys.

[27] Nakamura M, Matsuura M, Tezuka N, Sugimoto S, Une Y, Kubo H, Sagawa M (2013). Preparation of ultrafine jet-milled powders for Nd-Fe-B sintered magnets using hydrogenation–disproportionation–desorption–recombination and hydrogen decrepitation processes. Appl Phys Lett.

